# Comparative Evaluation of Stress Acting on Abutment, Bone, and Connector of Different Designs of Acid-Etched Resin-Bonded Fixed Partial Dentures: Finite Element Analysis

**DOI:** 10.3389/fbioe.2022.798988

**Published:** 2022-04-26

**Authors:** Saquib Ahmed Shaikh, Punith Rai, Sami Aldhuwayhi, Sreekanth Kumar Mallineni, Krishnapalli Lekha, Angel Mary Joseph, Vardharaj Vinutha Kumari, Roseline Meshramkar

**Affiliations:** ^1^ Department of Prosthodontics, College of Dentistry, Majmaah University, Majmaah, Saudi Arabia; ^2^ Department of Prosthodontics, SDM Dental College, Dharwad, India; ^3^ Department of Preventive Dental Science, College of Dentistry, Majmaah University, Majmaah, Saudi Arabia; ^4^ Center for Transdisciplinary Research (CFTR), Saveetha Institute of Medical and Technical Sciences, Saveetha Dental College, Saveetha University, Chennai, India

**Keywords:** finite element analysis, resin-bonded, partial denture, stresses, prosthodontic

## Abstract

**Background:** Finite element analysis (FEA) is one of the best methods for evaluating the stress distribution of restorations, such as fixed partial dentures. The development of resin cement has transformed prosthesis bonding and retention properties. Resin-bonded fixed partial dentures (RBFPD) have been considered minimally invasive treatment options for the prosthetic rehabilitation of single missing teeth.

**Objectives:** The aim of this study was to evaluate the stress load and distribution in four different designs of acid-etched RBFPDs using FEA.

**Materials and Methods:** The designs included standard tooth preparation principles and additional features. The first premolar and first molar abutments replaced the missing second premolar. Designs 1, 2, 3, and 4 included (1) lingual wings and occlusal rests; (2) wings and proximal slices; (3) wings, rests, and grooves; and (4) wings, rests, grooves, and occlusal coverage. The prepared models were restored with RBFPDs. A load of 100 N was applied to the central groove of the pontic to simulate occlusal forces. The materials used in the models were considered to be isotropic, homogeneous, and linearly elastic. FEA was used to reveal stresses acting on the abutment, bone, and connector in all prosthesis designs.

**Results:** The stresses transmitted to the abutment and bones were lowest for design 3, using wings, rests, and grooves. The stresses acting on the connector were the weakest in design 2. The stresses transmitted to the abutment and bone were highest in designs 1 and 4. The stresses transmitted to the connector were highest in design 3.

**Conclusion:** The wings, rests, and grooves design is possibly the ideal and conservative tooth preparation design to receive a posterior RBFPD. This design transmits less stress to the abutments and less bone resorption in the FEA. It is most likely to be successful in the clinical provision and ensures the longevity of the prosthesis.

## Introduction

Conventional resin-bonded fixed partial dentures (RBFPDs) offer a limited treatment option for prosthetic rehabilitation of a single edentulous space. With the advent of more contemporary designs and retention aids, RBFPDs can be incorporated into a broader range of edentulous positions (Tanaka et al., 2019). These include smaller-sized anterior teeth and premolars. The success of etched-cast RBFPDs is mainly dependent on tooth preparation (Arora et al., 2014). Various researchers have proposed designs with varying claims for enhancing retention and resistance form (Lin et al., 2005; Tanoue, 2016; Acharya et al., 2021). The standard preparations have wings and occlusal rests on the abutment teeth, while other patterns reportedly have proximal slices, grooves, and extensive coverage on occlusal surfaces. Nevertheless, clinicians have no general agreement about which design is best suited for a long-lasting service of RBFPDs (Lin et al., 2005). The conservative designs of RBFPD result in an excellent stress concentration in the abutment because of its geometry; the maximum stresses in restoration are known to be concentrated around the connector region (Tanoue, 2016). Excessive stress on the periodontal ligament leads to the resorption of supporting bone and weakening of the abutment teeth, leading to the restoration’s failure (Acharya et al., 2021). It becomes imperative to evaluate the stresses acting on the abutment, bone, and connector in various tooth preparation designs to determine the most suitable combination for enhancing the retention and clinical longevity of RBFPDs (Tanoue, 2016).

Finite element analysis (FEA) is a powerful tool to analyze the designed engineering parts for their strength. The developed features must be robust in design and sustainable against all kinds of loading conditions and work satisfactorily during their design life. For example, backhoe excavators work in severe working environments with cyclic operations. During the design stage, it is imperative to examine the strength of the various parts of the backhoe excavator for the maximum breakout force condition. This can be achieved through performing the FEA of all the parts of the backhoe excavator attachment. However, it is not enough to provide components with a safe design based on strength only, and it should also be light in weight, reliable, and economical. Structural weight optimization is the best solution to obtain the parts with reduced weight without compromising their strength. FEA is a quick and low-cost method to investigate stress distribution and strain patterns of complex restorations such as fixed partial dentures (FPDs) (Medina-Galvez et al., 2021). Many studies have compared the functional behavior of three-unit RBFPDs, two-unit cantilevered RBFPDs (Lin et al., 2005; Tanoue, 2016), and cantilevered RBFPDs (Acharya et al., 2021; Medina-Galvez et al., 2021). The three-dimensional assessments of the abutment teeth are necessary to accomplish more significant treatment outcomes using an RBFPD. Based on the three-dimensional information, clinicians would choose the types of frameworks, such as three- or two-unit RBFDPs supporting the tooth (abutment) axis, periodontal structure, and the possible preparation area. Few published studies reported using FEA in prosthodontics (Botelho, 2000; Zalkind et al., 2003; Aversa et al., 2009; Silva et al., 2009; Anweigi et al., 2020; Medina-Galvez et al., 2021), and among them, very few studies focused on the stress distribution of the components of RBFDs (Botelho, 2000; Chow et al., 2002; Zalkind et al., 2003). Understanding the mechanism of RBFPD failures and improved knowledge of the biomechanical behavior of these restorations is obligatory (Botelho et al., 2014; Robati Anaraki et al., 2019; Esquivel-Upshaw et al., 2020). Hence, there is a need to evaluate stress among the components of RBFDs. Therefore, the study assessed stress concentration and distribution on the abutment, bone, and connector in four different designs of acid-etched RBFPDs using FEA.

## Materials and Methods

**FIGURE 1:**
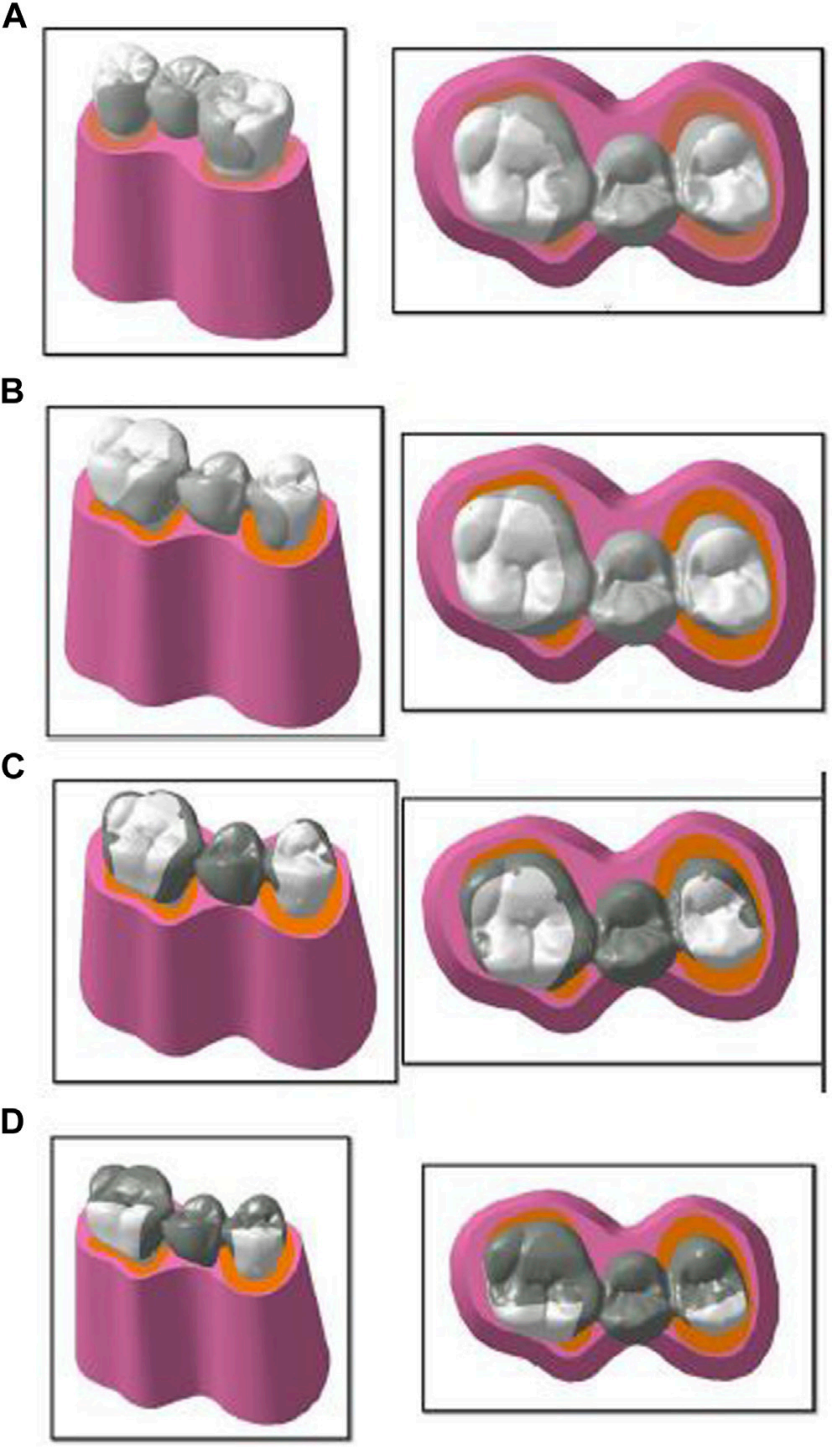
Geometry creation of four designs: **(A)** design 1 with lingual wings and occlusal rests; **(B)** design 2 with wings and proximal slices; **(C)** design 3 with wings, rests, and grooves; and **(D)** design 4 with wings, rests, grooves, and occlusal coverage used for finite element analysis.

A set of four models with four designs were prepared, in which the mandibular second premolar (pontic) was replaced using the mandibular first premolar and first molar as abutments. The abutments were prepared to receive the retainer of the RBFPDs. These retainer designs incorporated additional features along with standard tooth preparation principles. Design 1 (Figure 1) included lingual wings and occlusal rests distally on the premolar and mesially on the molar. A tooth reduction of up to 1 mm was made for wing preparation. Occlusal rests of the depth of 1 mm, buccolingual width of 2.5 mm, and mesiodistal width of 1.5 mm were prepared. In design 2 (Figure 1B), proximal slices began as an extension of the lingual wing preparation with a supragingival chamfer margin 0.5 mm occlusal to the gingival margin terminating 1.5 mm short of the facio-proximal line angle, without undermining the facial surface (Aversa et al., 2009). In design 3 (Figure 1C), along with the wings and proximal slice, grooves were added mid-lingually and proximally 1.5 mm short of the facio-proximal line angle, where the proximal slice preparation culminated. Grooves were prepared with a flat-end, tapered, fissured tungsten carbide bur up to a depth of 1 mm (Zalkind et al., 2003). In design 4 (Figure 1D), along with the wings, proximal slices, and grooves, the tooth had one occlusal coverage of 0.5 mm after a tooth preparation of 0.5 mm.

**FIGURE 2:**
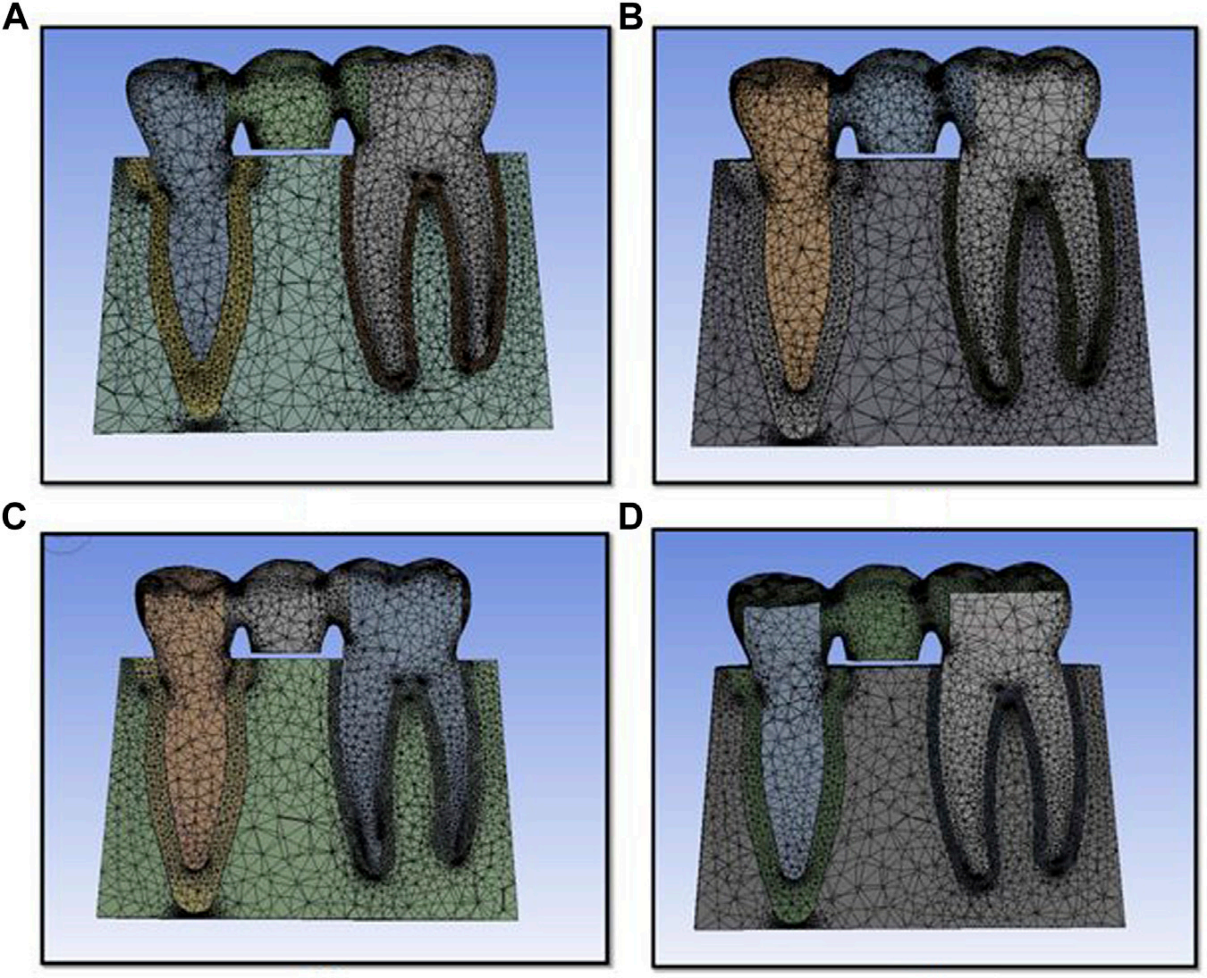
Finite element model used for the study for design 1 **(A)**, design 2 **(B)**, design 3 **(C)**, and design 4 **(D)**.

**FIGURE 3:**
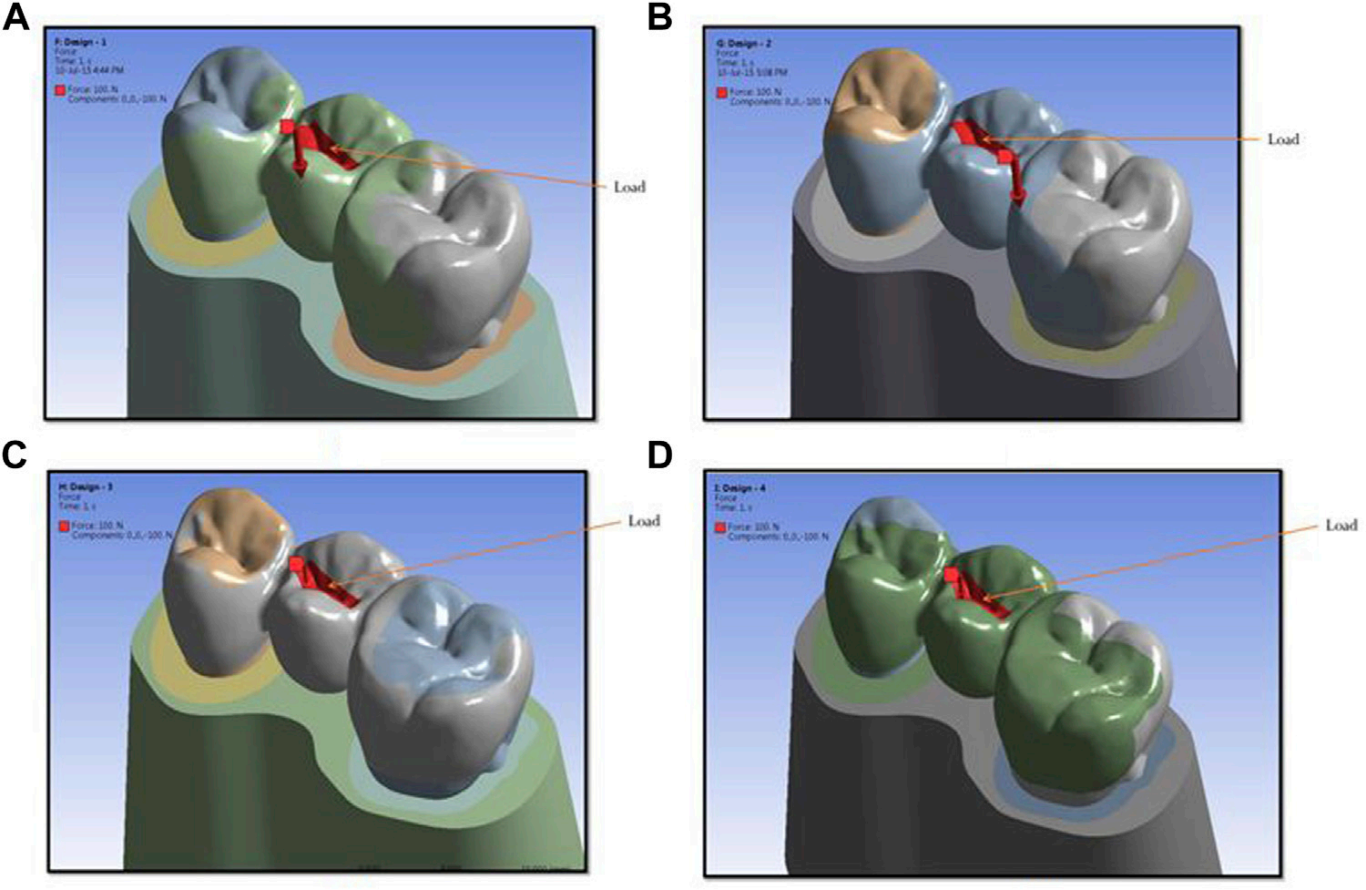
Showing load areas for design 1 **(A)**, design 2 **(B)**, design 3 **(C)**, and design 4 **(D)** used in the study.

The lingual cusps were involved in the preparation. The prepared teeth with wax patterns of the four designs were scanned using a computerized tomography scan. The tooth profile and profile of the restorations were obtained. This model scanning was made in a Dicom format file and converted to CAD (computer-aided design) geometry. This model was imported into the SOLIDWORKS software (Infid of Enhancing Engineering, India), and 3D models of the components were created and further edited (Figures 2, 3). Next, the meshing of the model (Figure 2) was carried out and divided into finite elements after importing it into ANSYS-11 workbench software. After meshing the components and maintaining the mesh criterion, the boundary conditions were determined to define the relationships between elements (Figure 3).

Boundary constraints were applied onto the inferior margin of the mandible and the mesial and distal ends of the prosthesis. Without boundary constraints, the stresses applied to the model will act infinitely. The following assumptions were made: (1) the material was assumed to be linear elastic, (2) the load remained constant concerning time, (3) other loads were neglected except for the applied load, (4) the boundary conditions do not change during loading, and (5) surface-to-surface contact between different parts is assumed to be of a “bonded” type. The contact area does not change during the analysis.

After meshing and maintaining the mesh criteria, material properties were applied to the model’s various components. The corresponding properties of various materials were obtained from the literature (Table 1) (Chow et al., 2002). The component was then run to obtain the stresses and contact analysis of the structure and the partial dentures’ respective components. After the analysis run was completed, the stresses in the system were visualized and noted down. A vertical load of 100 N was applied to the pontic’s central groove (El Salam Shakal et al., 1997). Four types of analysis were conducted with a constant load (Figure 5). The model was fully constrained at the bottom of the abutment teeth. In post-processing, the stress concentration zones were analyzed and tabulated. The 3D model of the dental structure and prosthesis was generated using CATIA V5 package as per the given specifications. The model was imported into Ansys Workbench; the 3D component can mesh as a finite element body utilizing this software. Material properties are described as per the specifications, and the components were subjected to loading and boundary conditions. Static structural analysis was carried out to find stress and deformation in various components. The principal stress in megapascals (MPa) and displacement in millimeters (mm) as well as locations and magnitudes were identified and used to evaluate biomechanical behavior. Maximum principal stress describes the highest stress, and minimal stress describes the lowest stress and can be considered tensile stress.

**TABLE 1:** Material properties used in the study.

Material	Component	Young’s modulus (MPa)	Poisson’s ratio
Dentin	Abutment	18,600	0.31
Bone	Cortical bone	15,000	0.3
Soft Bone	Cancellous Bone	1,500	0.3
Cobalt chromium alloy	Connector	218,000	0.3

## Results

**FIGURE 4:**
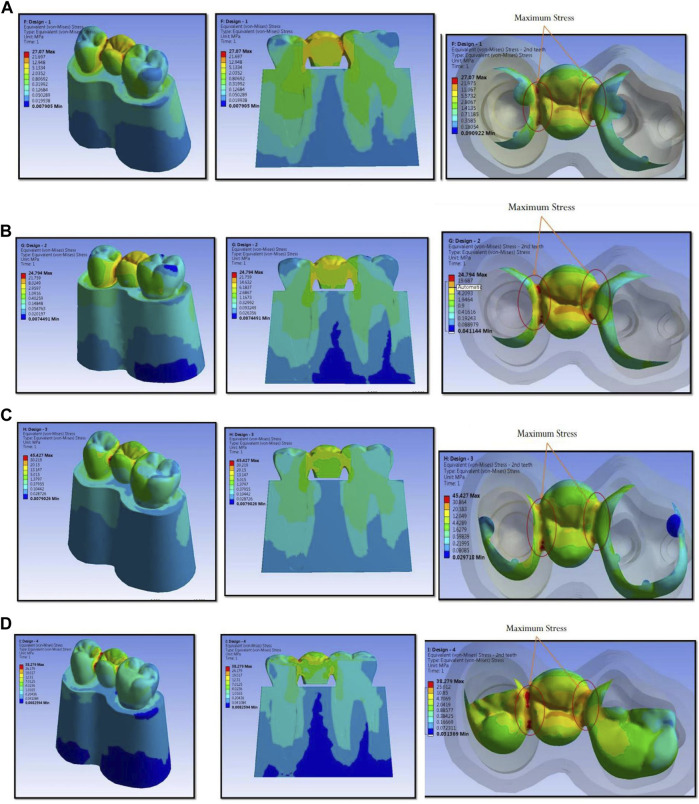
Maximum stress observed is in prosthesis and the magnitude and stress concentration in prosthesis for design 1 **(A)**, design 2 **(B)**, design 3 **(C)**, and design 4 **(D)** used in finite element analysis.

**FIGURE 5:**
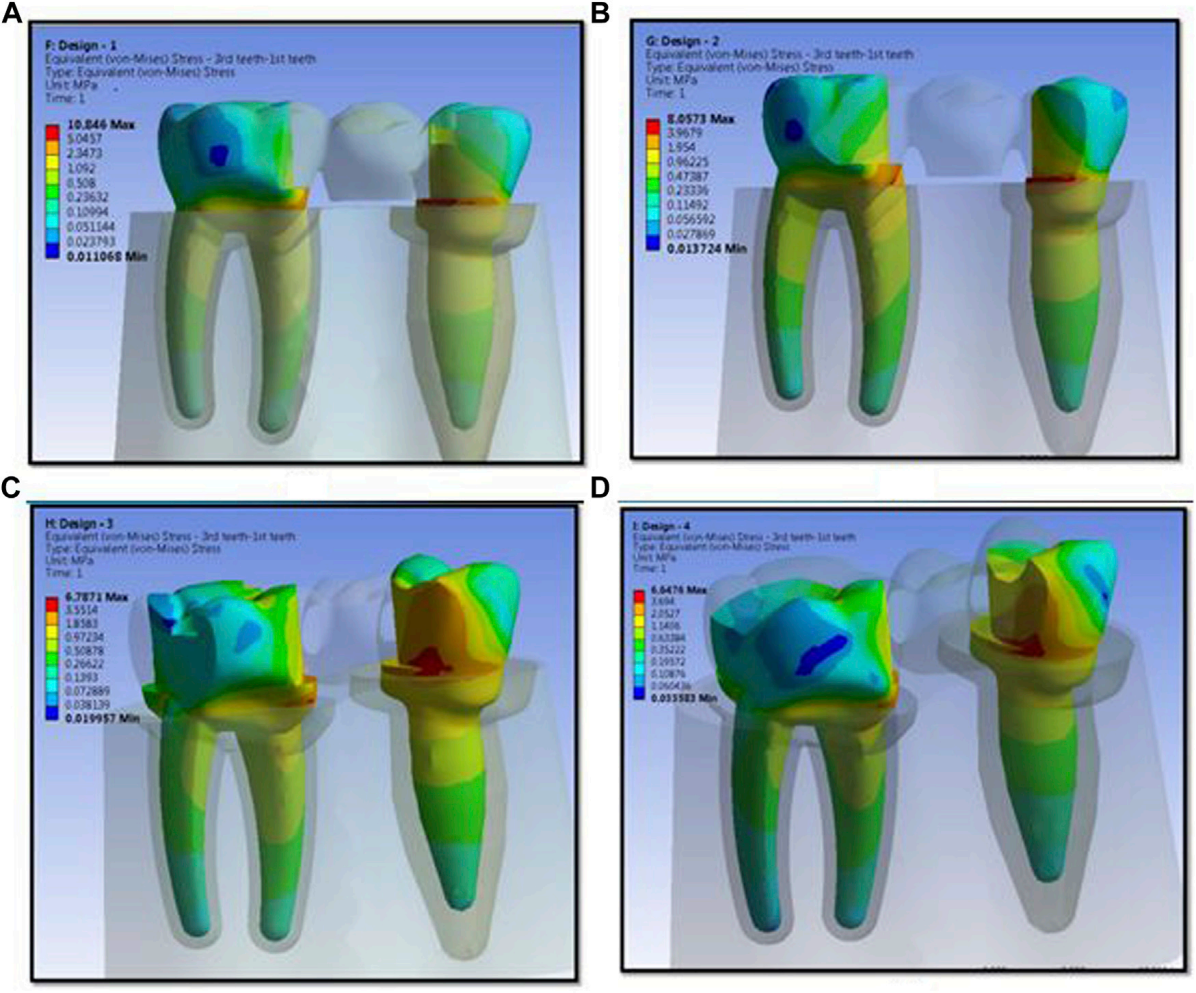
Stress distribution on abutments for design 1 **(A)**, design 2 **(B)**, design 3 **(C)**, and design 4 **(D)** used in finite element analysis.

**FIGURE 6:**
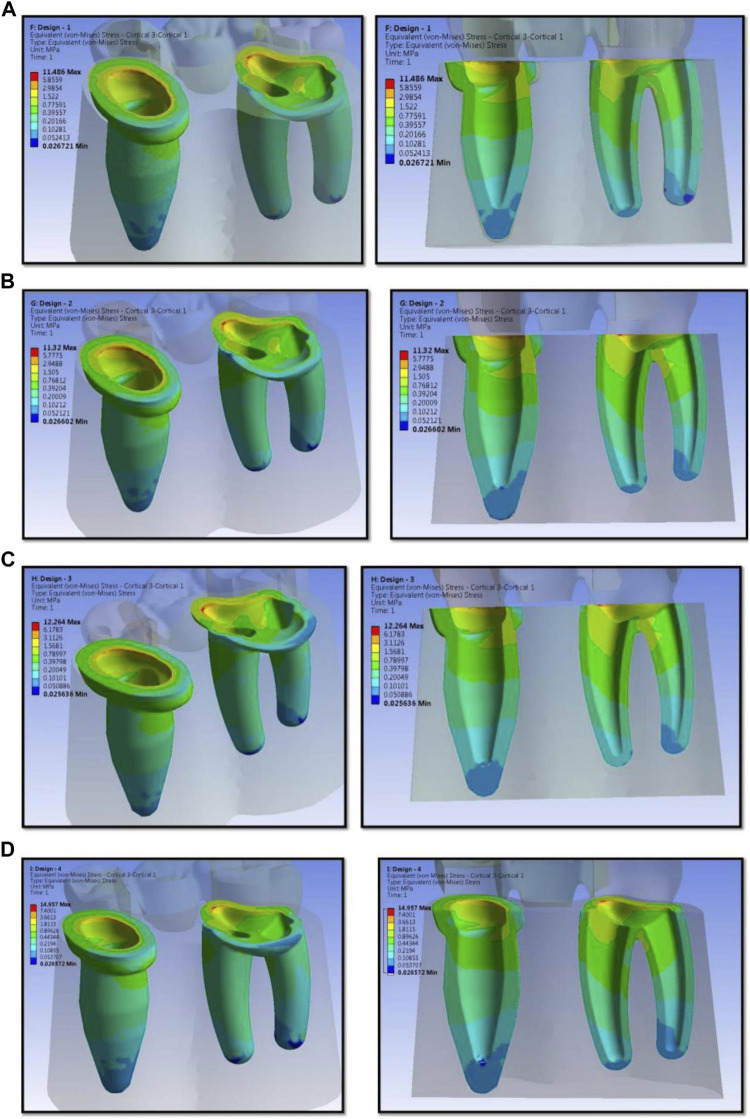
Stress distribution on the different designs of resin-bonded fixed dental prosthesis on cortical bone for design 1 **(A)**, design 2 **(B)**, design 3 **(C)**, and design 4 **(D)** used in finite element analysis.

**FIGURE 7:**
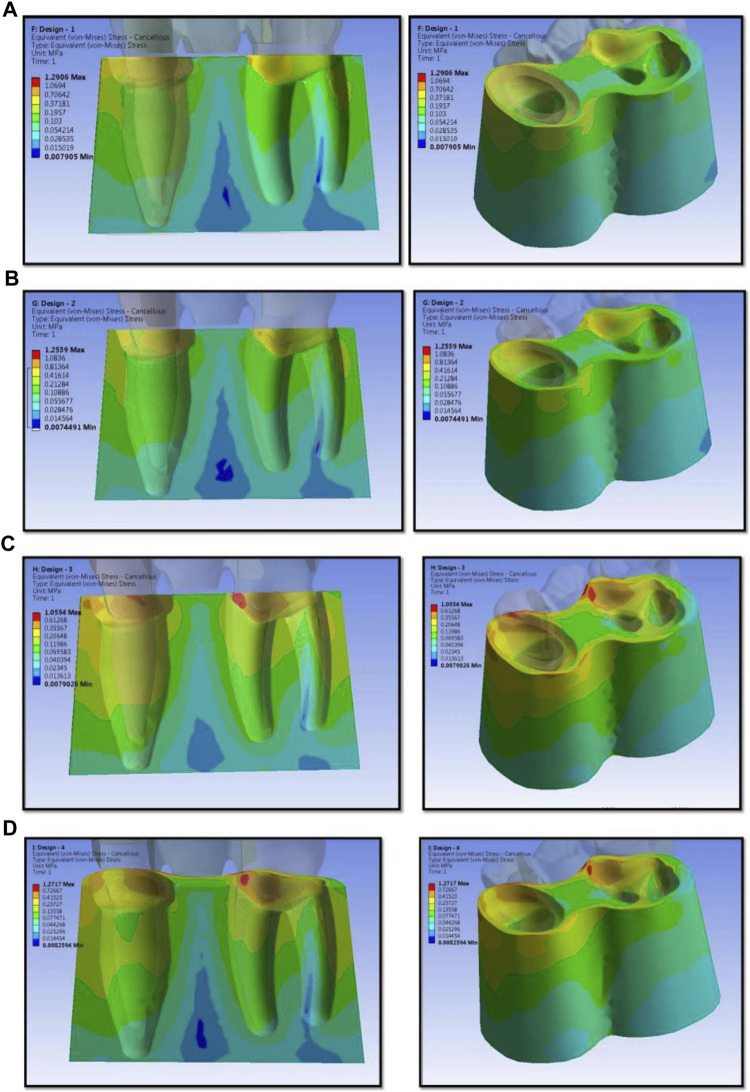
Stress distribution on cancellous bone for design 1 **(A)**, design 2 **(B)**, design 3 **(C)**, and design 4 **(D)** used in finite element analysis.

**FIGURE 8:**
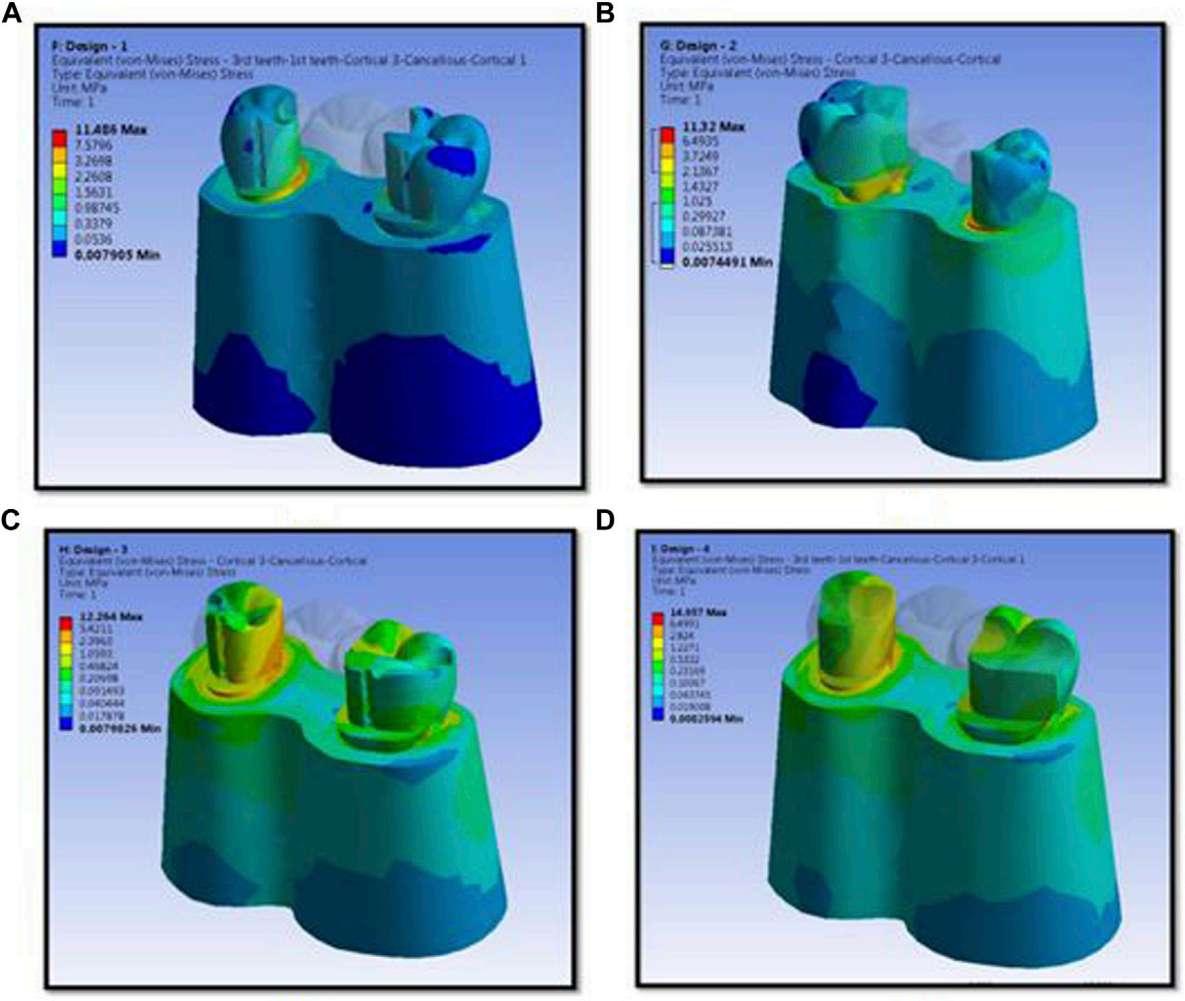
Stress distribution in cortical bone, cancellous bone, and teeth for design 1 **(A)**, design 2 **(B)**, design 3 **(C)**, and design 4 **(D)** used in finite element analysis.

**FIGURE 9:**
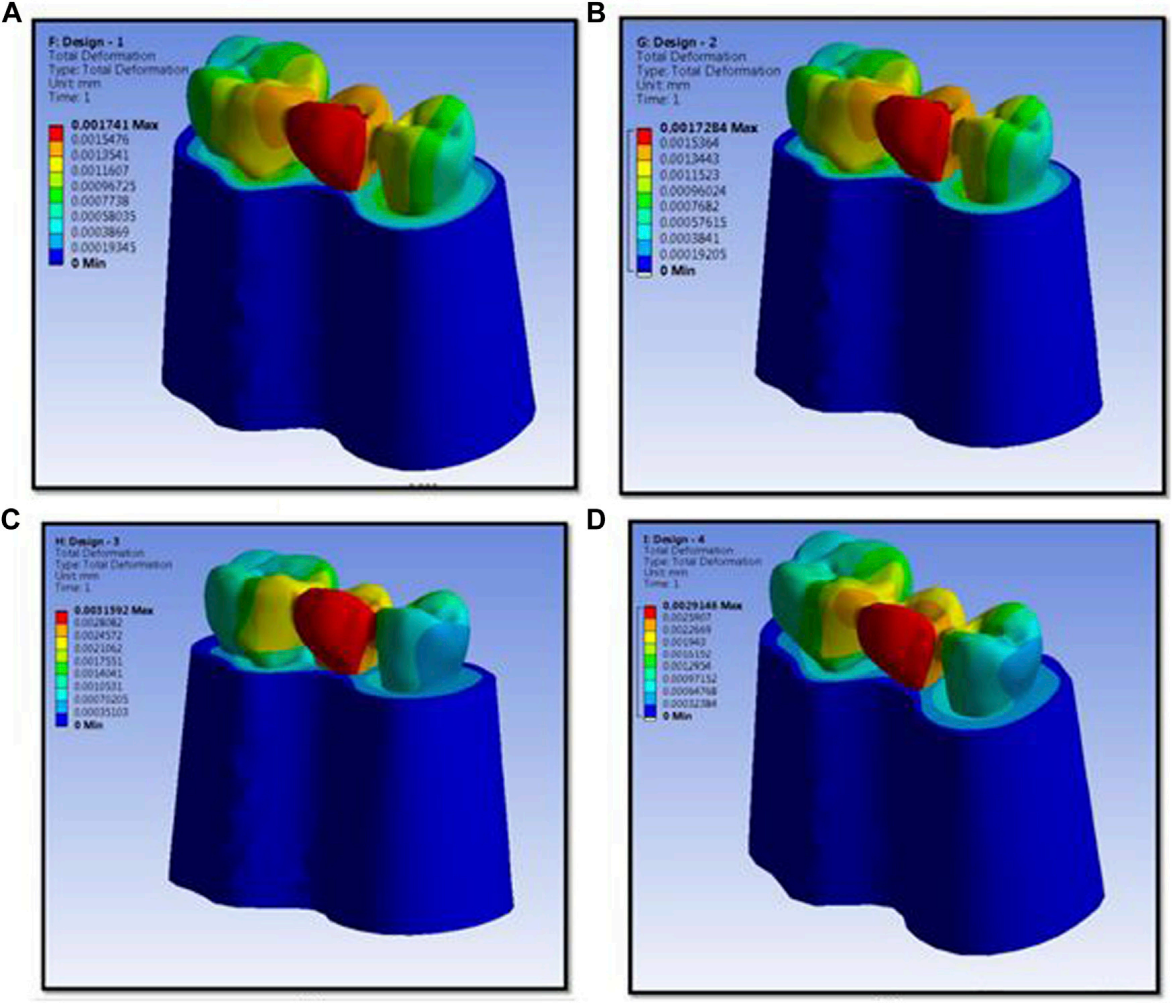
Maximum deformation observed in prosthesis and the magnitude for design 1 **(A)**, design 2 **(B)**, design 3 **(C)**, and design 4 **(D)** used in finite element analysis.

**FIGURE 10:**
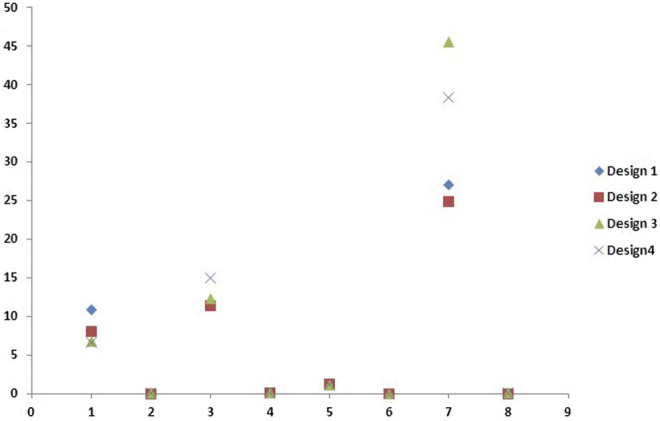
Scatter diagram showing distribution stresses (in megapascals) on various components of different designs studied in the study.

The numerical analysis for Von Mises stresses occurring for the FEM models is summarized in Table 2. The maximum stress utilized for design 3 was the highest (45.427 MPa), followed by design 4 (38.279 MPa), design 1 (27.07 MPa), and design 2 (24.794 MPa). The maximum stress observed was in the prosthesis, and the magnitude among the study designs is shown in Figure 4. Design 3 showed the lowest stress on the abutment (6.7871 MPa); likewise, design 4 exhibited low stresses (6.6476 MPa), nearly the same as those on design 3. Both designs had more tooth surface coverage; hence, lower stresses acted on the abutment. Design 1 showed the highest stress (10.846 MPa) on the abutment among all four designs. The stress distribution on teeth is shown in Figure 5. It could be attributed to the rest as predominantly responsible for transferring stresses from the pontic to the abutment. Design 3 transferred the least amount of stress (11.24 MPa), while designs 1 (11.24 MPa), 2 (11.24 MPa), and 4 (14.95) transferred higher stresses onto the cortical bone (Figure 6). Similarly, design 3 transferred the least amount of stress (1.055 MPa), while designs 1 (1.29 MPa), 2 (1.25 MPa), and 4 transferred higher stresses (1.2717 MPa) onto the cancellous bone (Figure 7). The stresses acting on the connector were lowest in design 2 due to proximal extensions, which reduced the stress within the RBFPD. Design 2 (27.07 MPa) showed low stresses on the connector, almost comparable to design 1 (24.794 MPa). Higher stresses on the connector were observed in designs 3 and 4 due to the increased coverage area of the prosthesis on abutment teeth (Figure 8). Maximum deformation was observed in the prosthesis, a magnitude less for design 4 (0.0029146 mm) followed by design 3 (0.0031592 mm), design 2 (0.00172 mm), and design 1 (0.001741 mm). The prosthesis had a larger share of the total load, resulting in stresses in these designs (Figure 9). The distribution stresses on various components of the study’s different designs are shown in the scatter diagram (Figure 10).

**TABLE 2:** Comparison of stress distribution on the abutment, cortical bone, cancellous bone, and connector in all designs of resin-bonded fixed partial dentures.

Design	Abutment (MPa)	Cortical bone (MPa)	Cancellous bone (MPa)	Connector (MPa)
1	10.84	11.48	1.29	27.07
2	8.05	11.32	1.25	24.79
3	6.24	11.24	1.05	45.42
4	6.64	14.95	1.27	38.27

## Discussion

RBFPDs were first described in the 1970s and have evolved significantly (Medina-Galvez et al., 2021). The development of resin cement has revolutionized prosthesis retention and bonding. RBFPDs are reversible and minimally invasive treatment options for prosthetic rehabilitation of single missing teeth (Aversa et al., 2009; Silva et al., 2009; Acharya et al., 2021). They also serve a masticatory and aesthetic function without compromising the abutment (Zalkind et al., 2003; Aversa et al., 2009; Silva et al., 2009; Anweigi et al., 2020). This is important for young patients who may have endodontic complications following extensive tooth preparation. Despite the advantages, the use of RBFPDs as definitive restorations remains controversial due to a lack of long-term prospective data on prosthetic success. A recent systematic review has estimated 5-year survival rates of 87.7% for resin-bonded prostheses and greater than 90% for conventional fixed prostheses depending on the design (Lin et al., 2003).

Although there is a lower than 94.5% success rate reported for implant-retained single crowns over the same 5-year follow-up, resin-bonded bridgework has the advantages of being less invasive and requiring a shorter total treatment time and less financial commitment. Since the components in fixed prosthesis–bone systems are complex geometrically, FEA is the most suitable tool for analyzing them (Borcic et al., 2007). A key factor for the success or failure of a dental prosthesis is how stresses are transferred to the surrounding bone. FEA helps us predict the pattern, concentration, and stresses acting on the prosthesis and surrounding tissues. This helps us determine the failure mechanisms of the prosthesis. Excessive stresses acting on the surrounding bone and tissues cause tissue injury. The body then attempts to repair the injury and periodontium. This can occur if the forces are diminished or the tooth drifts away. The periodontium is remodeled to cushion its impact if the offending stress is chronic. This causes a widening of the periodontal ligament space due to the resorption of bone. It also results in angular bone defects without periodontal pockets and causes mobility of teeth. Subsequently, it might lead to the restoration’s failure due to the loss of the abutment tooth (Della Bona et al., 2013). Furthermore, undue stresses acting on the connector region can lead to fractures. The adequate thickness of the connector area is essential to prevent flexing and fracture, and a minimum thickness of 0.8 mm is recommended for fracture resistance (Pjetursson et al., 2007). The early design of the posterior Maryland Bridge included axial coverage and an occlusal rest. There was little proximal and lingual enamel preparation.

Posterior RBFPDs appeared to require a 180-degree-plus circumferential preparation of abutment teeth for predictable success (Toman et al., 2015). The design preparation was modified by incorporating mechanical retention features such as grooves for resistance. The L-shaped retainer covered one-half of the lingual cusps with a groove at the far side of the buccal line angle and another groove at the far opposite side to hold the abutment teeth firmly. Chow et al. presented an approach using a groove, a plate, and a strut, which involved minimal preparation of the posterior abutment to receive an RBFPD using a base metal alloy (Modi et al., 2015). Botelho (2000) recommended that the major retainer has a wraparound configuration on at least three abutment surfaces or has strategically placed opposing axial grooves or slots for long-span prostheses to replace two or more missing teeth. Another report described a methodical preparation for posterior partial veneered restorations that provided proper posterior occlusal function and isolated the enamel’s occlusal contact area to maintain the occlusion’s vertical dimension (Saad et al., 1995).

A long-term follow-up indicated that abutment teeth mobility is one of the decisive prognostic factors for the success of RBFPDs (Farshad et al., 2013). Furthermore, it was reported that an RBFPD without any retentive preparation design failed significantly at a higher rate (Sichi et al., 2021). Saad et al. suggested that the addition of retentive grooves made a statistically significant difference in resistance to debonding forces (Saad et al., 1995). The results demonstrated that design modifications were necessary to improve clinical longevity. Therefore, the application of resin-bonded retainers with additional retentive structures, such as a pinhole and grooves in the anterior region, was recommended. In addition to this, the retainer thickness also affects the retention of the prosthesis. Chun et al. concluded that a minimum retainer thickness of 0.8 mm was required for long-term RBFPD success (Pjetursson et al., 2007). Very few studies have assessed stress acting on various designs of RBFPDs. The present study was aimed to evaluate a design that transmits less stress to the prosthesis and supporting structures. In the present study, four different RBFPD designs for tooth preparation included retentive features such as rests, grooves, and occlusal coverage. The tooth preparation for the retainers incorporated all design patterns currently used for etched-cast RBFPDs. Design 1 was the conventional RBFPD design with lingual reduction and occlusal rests. Design 2 combined slices, which gave proximal extensions for the RBFPDs. The proximal extensions reduced stress around the connector and the rest areas. Design 3 consisted of grooves in addition to the rests, wings, and slices. The grooves provided additional retention against displacing and rotational forces (Botelho, 2000). Proximal grooves compensate for the lack of proximal wraparound, limited by aesthetic requirements. Vertical grooves were a particular feature that has been identified as reducing stresses on the cement bond and increasing resistance to debonding forces. The preparation involves irreversible damage to abutment teeth, and even when minimal preparation is intended, dentin exposure is likely during preparation. Design 4 added occlusal coverage to the features mentioned above. Occlusal coverage has improved the retention and resistance form of RBFPDs (Lin et al., 2005; Arora et al., 2014). In the present study, a vertical force of 100 N was applied to the pontic’s natural groove, about one-sixth of the maximum biting force (El Salam Shakal et al., 1997; Zalkind et al., 2003). This region was chosen since it occludes with the stamp cusp of the opposing tooth. The resultant stresses acting on each area of the four models were assessed and tabulated. In all designs, the maximum stresses on the prosthesis were seen in the area around the connector. The maximum stresses were concentrated at the cervical margin of the abutment margin around the pontic on the teeth. It can be seen that stresses were highly concentrated near the connector but were relatively uniformly stressed throughout the occlusogingival direction away from the connector sites. Design 1 is the conventional design used by the majority of clinicians. Lesser stress concentration on the connector makes it less susceptible to fracture. Nevertheless, high stresses are transmitted onto the abutment and bone, which can be attributed to the rests of the design. Design 2 showed the least amount of stress on the connector, but owing to the high stresses transmitted onto the abutment and bone, it becomes a less-preferred design of RBFPDs. Design 3, despite the high stress within the connector, transmits the least amount of stress into the abutment and bone. As a result, there is less bone resorption and no harm to the supporting structures, resulting in the least likelihood of failure of RBFPD. The grooves included in the design increase the surface area present for bonding, adding to the retentive ability of the prosthesis (AlShaarani et al., 2019). These features ensure the clinical longevity of the prosthesis. Design 4 offers added coverage for the prosthesis but transmits high stresses to the abutment and bone. It is a nonconservative design of tooth preparation. Additionally, high stresses act on the connector, which may increase fracture probability in this region. These factors make design 4 unsuitable for RBFPDs.

A recent systematic review (Reddy et al., 2019) suggested that FEA analysis enhances the validity of the models. A Swedish study (Bakitian et al., 2020) recommended that the framework designs play a significant role in stress distribution. A Japanese study (Tsouknidas et al., 2020) reported that ceramic veneers could restore the biomechanical behavior of prepared anterior teeth. In the present study, stress load was evaluated on the connector, abutment, and bone of four different RBFPDs. Posterior teeth were used for evaluation in the present study. The authors found that the use of wings, rests, and grooves was ideal for tooth preparation to receive RBFPD; it was evident that this was a more conservative design than other designs studied. FEA was not a practical method of assessing the failure of the restorations, and many different clinical situations need to be considered. The properties of dentin were considered for the whole tooth, and the periodontal ligament’s role was not considered a potential limitation of the present study. The methodology of the present study would offer the possibility of stress analysis in the restorative material, primarily. A stress analysis was not carried out in the present study, and it was not our objective; hence, this is also considered a limitation.

The FEA can provide detailed quantitative data at any location within the mathematical model. Thus, FEA has become a valuable analytical tool in assessing various dentistry systems (Pjetursson et al., 2008). Keulemans et al. (2015) used five framework materials (zirconia, glass-ceramic, gold alloy, indirect fiber-reinforced composite, and direct fiber-reinforced composite). They reported that there were differences in biomechanical behavior between various RBFPDs. The authors concluded that an RBFPD made of FRC achieved promising stress distribution. These findings were not comparable with the present study since the study designs used in the present study were different from the designs used by Keulemans et al. (2015). Various researchers investigated anterior RBFPDs exceptionally with regard to design and loading. The use of RBFPDs in the posterior region is less predictable than in the anterior region because of occlusal forces. However, there is a lacuna in the published evidence assessing the factors associated with success in replacing teeth with RBFPDs in the posterior region (Keulemans et al., 2015; King et al., 2015).

In the present study, design 3 was found to have transmitted the least stress through and around the abutment teeth, and this results in lesser bone resorption around the abutment teeth. The study also found that the stress acting on the connector area was minimal in design 3. Reducing the stress acting in this region reduces the likelihood of fracture occurring in the restoration and increases the longevity of the restoration. Based on the above findings, design 3 is the design that should be advocated in clinical practice because it has better retention compared to other designs. The limitations of the present study include the following: (1) FEA was not a practical method for assessing the failure of the restorations, and many other clinical situations need to be considered; (2) the properties of dentin were considered for the whole tooth; and (3) the role of the periodontal ligament was not considered. The present study within these limitations established the RBFPDs in the posterior region by using FEA. Although stress concentrations often appear in FEA, the linear assumption used in a linear FEA does not precisely depict what is occurring in areas of stress. It is very unusual to fail as a fundamental component based on a stress concentration in a linear analysis.

## Conclusion

Within the limitations of this study, it can be concluded that the design with wings, rests, and grooves is possibly the ideal and conservative design of tooth preparation for receiving a posterior RBFPD. The design with wings, rests, and grooves transmits less stress to the abutments and results in lesser bone resorption. It is most likely to be successful in clinical service, ensuring the longevity of the prosthesis.

## Data Availability

The raw data supporting the conclusions of this article will be made available by the authors, without undue reservation.
