# Corrigendum: Comparative Evaluation of Stress Acting on Abutment, Bone, and Connector of Different Designs of Acid-Etched Resin-Bonded Fixed Partial Dentures: Finite Element Analysis

**DOI:** 10.3389/fbioe.2022.978067

**Published:** 2022-07-18

**Authors:** Saquib Ahmed Shaikh, Punith Rai, Sami Aldhuwayhi, Sreekanth Kumar Mallineni, Krishnapalli Lekha, Angel Mary Joseph, Vardharaj Vinutha Kumari, Roseline Meshramkar

**Affiliations:** ^1^ Department of Prosthodontics, College of Dentistry, Majmaah University, Riyadh, Saudi Arabia; ^2^ Department of Prosthodontics, SDM Dental College, Dharwad, India; ^3^ Department of Preventive Dental Science, College of Dentistry, Majmaah University, Riyadh, Saudi Arabia; ^4^ Center for Transdisciplinary Research (CFTR), Saveetha Institute of Medical and Technical Sciences, Saveetha Dental College, Saveetha University, Chennai, India

**Keywords:** finite element analysis, resin-bonded, partial denture, stresses, prosthodontic

In the published article, there was an error in affiliations **1** and **3**. Instead of “^1^Department of Prosthodontics, College of Dentistry, Majmaah University, Riyadh, Saudi Arabia; ^3^Department of Preventive Dental Science, College of Dentistry, Majmaah University, Riyadh, Saudi Arabia,” it should be “^1^Department of Prosthodontics, College of Dentistry, Majmaah University, Majmaah, Saudi Arabia; ^3^Department of Preventive Dental Science, College of Dentistry, Majmaah University, Majmaah, Saudi Arabia.”

Furthermore, there was an error in the **Funding** statement. The correct Funding statement appears below:

